# Insights into sarcopenia awareness among trainee physicians: a survey-based analysis of a university hospital, Thailand

**DOI:** 10.2478/abm-2025-0003

**Published:** 2025-02-28

**Authors:** Manchumad Manjavong, Panita Limpawattana, Khanyanut Ojongpien, Nutwara Saengwijit, Prapassawan Tanlawan

**Affiliations:** Medicine Department, Faculty of Medicine, Khon Kaen University, Khon Kaen 40002, Thailand

**Keywords:** attitude and perception toward aging/aged, disability, frailty, health personnel, knowledge, survey design

## Abstract

**Background:**

Sarcopenia is associated with a decline in functionality and disability among older adults. Extensive research has been conducted on the subject matter of sarcopenia; nevertheless, there is a paucity of studies documenting the extent to which practicing physicians are integrating sarcopenia into their clinical practice in the Asian context.

**Objectives:**

To examine trainee physicians' attitude and knowledge in sarcopenia and factors associated with higher scores on knowledge in sarcopenia.

**Methods:**

An electronic survey of trainee physicians of the Faculty of Medicine, Khon Kaen University, Thailand was conducted from November 2022 to December 2023. The survey consisted of questionnaires designed to assess both attitude and knowledge toward sarcopenia. All trainees were invited to participate, and the completed questionnaires were subsequently returned to the researchers for analysis.

**Results:**

A total of 211 trainees enrolled; most of them were familiar with “sarcopenia” (70.1%) but lack of confidence in diagnosis, prevention, and management. The total scores on general knowledge about sarcopenia were 22 out of 30 (73.3%). They scored well in the area of “etiology” (75%) and “terminology and importance” (70%) but fair in “diagnosis” (62.5%) and “management and prevention” (62.5%). The lower age of the trainee physicians, increased years of practice, and “training in internal medicine” were associated with higher scores.

**Conclusions:**

Trainee physicians were aware of the term “sarcopenia” but had limited knowledge in diagnosis, prevention, and management. Certain factors related to better scores on the knowledge evaluation were identified.

Sarcopenia constitutes a geriatric syndrome characterized by a gradual and widespread dysfunction of the skeletal muscle, which exhibits an augmented prevalence as individuals age [1,2,3]. In general, sarcopenia affects about 10%–16% of older adults [4]; however, the prevalence of this condition varies depending on the studied population and diagnostic criteria. For instance, the prevalence was 47.9% among hospitalized patients aged ≥65 years in neurological rehabilitation departments [5], and it was 53.8% in patients aged ≥75 years admitted for hip fracture [6]. This particular condition is linked to various unfavorable health consequences such as compromised physical mobility, heightened morbidity, and mortality. Furthermore, it is connected to the accelerated advancement of cardiovascular ailments, incidents of falling, extended periods of hospitalization, fractures, perioperative complexities, and a decline in quality of life [3, 7, 8]. The primary underlying factor leading to sarcopenia is an imbalance between anabolic and catabolic muscle homeostasis, which may or may not be accompanied by neuronal degeneration. The process of aging, chronic diseases, malnutrition, and lack of mobility all play a role in the progression of this condition [9]. Sarcopenia was attributed a distinct code in the International Statistical Classification of Disease and Related Health Problems in the year 2016. This development spurred global efforts to conduct diagnostic and therapeutic trials, aiming to enhance recognition and understanding of this particular medical condition [1]. Originally, the identification of sarcopenia relied solely on the assessment of diminished muscle mass. However, various global research collectives have progressively shifted their attention toward muscle function, encompassing both muscular strength and physical performance. This shift in focus has been motivated by the predictive nature of these measures in terms of patient outcomes, as well as their responsiveness to therapeutic interventions, as demonstrated by several studies such as the Asian Working Group on Sarcopenia [1], the Korean Working Group on Sarcopenia (KWGS) [10], the European Working Group on Sarcopenia for Older People (EWGSOP) [11], and the Australian and New Zealand Society for Sarcopenia and Frailty Research (ANZSSFR) [12].

Despite the increasing amount of research conducted on sarcopenia and the publication of numerous practice guidelines, the available studies on the attitude and knowledge toward sarcopenia are limited [1, 10,11,12]. The existing results indicate that the awareness and knowledge among physicians regarding sarcopenia vary greatly. Several studies have reported a lack of awareness and knowledge specifically in relation to diagnostic criteria, clinical application, and management of sarcopenia [13]. For example, a study conducted among Dutch physicians revealed that only a small proportion of them incorporated diagnostic measures into their practice, although most of them were familiar with the concept of sarcopenia [14]. Another study conducted in Australia and New Zealand showed that the healthcare professionals had limited knowledge of the diagnostic algorithm for sarcopenia [15]. Similarly, a study in the United States found that while the term “sarcopenia” was familiar to geriatricians and physical medicine and rehabilitation specialists, a small percentage of internists and family physicians were familiar with it [6].

The existing literature on the correlation between attitude and sarcopenia among physicians in the Asian context is insufficient. The results of the study should emphasize the significance of enhancing physicians' awareness and knowledge to effectively diagnose, prevent, and manage sarcopenia. Moreover, it would be advantageous to comprehend the obstacles that hinder awareness and knowledge regarding sarcopenia to implement appropriate interventions. Thus, the objective of this study was to assess the attitude and knowledge pertaining to sarcopenia among trainee physicians, as they are the primary healthcare providers who interact with patients and spend more time with them compared with specialized staff. Furthermore, this study aimed to identify the factors associated with possessing a higher level of knowledge about this condition.

## Methods

### Participants and study design

According to the guidelines provided by the Asian Working Group for Sarcopenia: 2019 consensus update on sarcopenia diagnosis and treatment (AWGS 2019) [1], the electronic questionnaires were developed to evaluate individuals' attitudes and knowledge pertaining to sarcopenia. The process of constructing these questionnaires involved a deliberate and systematic approach. The questionnaires encompassed various aspects of sarcopenia, including its definition, significance, causes, diagnosis, management, and prevention. They consisted of two primary sections: (1) gathering demographic data and exploring prior experience in the fields of geriatric medicine or nutrition training and (2) assessing attitudes toward sarcopenia (consisting of 9 items) as well as general knowledge about sarcopenia (comprising 30 items, which were further divided into 14 items related to terminology and importance, 4 items pertaining to etiology, 8 items focusing on diagnosis, and 8 items addressing management and prevention). These items were evaluated using a 5-point Likert scale. The details of the questionnaires were described elsewhere [16].

The electronic questionnaires were disseminated to all trainee physicians who cared for geriatric patients from any specialties at Srinagarind Hospital, Faculty of Medicine, Khon Kaen University, Thailand from November 2022 to December 2023. This hospital is a university and tertiary care hospital in the northeastern part of Thailand. The trainee physicians were requested to diligently fulfill the questionnaire at their discretion. Confidentiality was ensured, and no forms of incentive were provided. Subsequently, the accomplished surveys were returned to the investigators.

The Khon Kaen University Institutional Review Board approved this study (reference No. HE 651502). The Ethics Committee determined this study as exempt category and approved the final protocol where participants were informed about the study, but written consents were not required, based on the criteria laid out in the university announcement (No. 2178.2563) regarding the survey procedure. Therefore, the requirement for informed consent was waived.

### Statistical analysis

The questionnaires' content validity was evaluated using the scale-content validity index (S-CVI) by a geriatrician and two physician nutrition specialists, which yielded a score of 0.94. Face validity was also assessed with the target participants. Descriptive statistics were employed to analyze the demographic data, which were then presented as percentages, means, and standard deviations. In cases where the data did not adhere to a normal distribution, medians, and interquartile ranges were utilized instead. The factors associated with general knowledge of sarcopenia scores were examined through stepwise forward multiple regression analysis with logistic transformation. Responses expressing agreement with the statements were consolidated into two categories: strongly agree and agree. All other responses were classified as disagreeing. Statistically significant differences were determined by a *P*-value <0.05. The data analysis was conducted using STATA version 10.0 (StataCorp).

## Results

### Characteristics of the participants

A total of 211 electronic questionnaires were submitted by the trainee physicians, resulting in a response rate of 42.2% (211 out of 500 participants). The characteristics of those respondents are displayed in **Table 1**. The median tenure as qualified physicians was 2 years. The trainee physicians were mainly trained from the Department of Internal Medicine, followed by surgery and orthopedics. A small fraction of them had received training in geriatric medicine and nutrition.

**Table 1.** Characteristics of studied population

**Variables**	**n = 211**	
Age (years); med (IQR 1, 3)	26	(25, 29)
Male; n (%)	108	(51.2)
Duration of practice (years); med (IQR 1, 3)	2	(0.9, 3.3)
Working place		
Internal medicine	82	(38.8)
Surgery	37	(17.5)
Orthopedics	13	(6.1)
Family medicine	12	(5.6)
Physical medicine and rehabilitation	6	(2.8)
Others	61	(31.2)
Clinical experience of training	48	(23.8)
Geriatric medicine	24	(11.4)
Nutrition	42	(19.9)

GP, general practitioner; IQR, interquartile range; n, numbers of participants; med, median.

### Attitude regarding sarcopenia among trainee physicians

The attitude of trainee physicians regarding sarcopenia is illustrated in **Table 2**. Most of them had known about “sarcopenia,” albeit lacking proficiency in diagnosing this particular condition. Almost 50% of the trainee physicians concurred and strongly concurred that the prevention of common medical ailments holds greater significance than the prevention of sarcopenia. Conversely, the majority of the respondents expressed disagreement and strong disagreement with the notion that sarcopenia is unrelated to their patients, likening it to cachexia and starvation. Approximately 60% of the participants agreed and strongly agreed that all hospitalized individuals should undergo formal physical therapy consultation if their anticipated stay exceeds 1 week.

**Table 2.** Attitude on selected issues of sarcopenia among trainee physicians

**Statement**	**Level of agreement (N = 211)**				

	**Strongly agree N (%)**	**Agree N (%)**	**Neutral N (%)**	**Disagree N (%)**	**Strongly disagree N (%)**
I have heard about “sarcopenia” before.	98 (46.4)	50 (23.7)	38 (18.0)	17 (8.1)	8 (3.8)
I know how to diagnose sarcopenia.	12 (5.7)	46 (21.8)	81 (38.4)	45 (21.3)	27 (12.8)
In clinical practice, the prevention of common medical diseases with severe complications, such as diabetes mellitus, and hypertension is more important than the prevention of sarcopenia.	39 (18.5)	65 (30.8)	57 (27.0)	31 (14.7)	19 (9.0)
Sarcopenia lies beyond the realm of my expertise, as it lacks any association with my patients.	14 (6.6)	27 (12.8)	40 (19.0)	65 (30.8)	65 (30.8)
Sarcopenia is like cachexia or starvation.	9 (4.3)	33 (15.6)	62 (29.4)	57 (27.0)	50 (23.7)
The patient without muscle atrophy is likely to have normal muscle mass.	5 (2.4)	37 (17.5)	52 (24.6)	70 (33.2)	47 (22.3)
The patient with normal muscle power is likely to have normal muscle mass.	18 (8.5)	60 (28.4)	63 (29.9)	47 (22.3)	23 (10.9)
Every hospitalized patient who is expected to stay over a week should have a physical therapy consultation.	48 (22.8)	88 (41.7)	49 (23.2)	24 (11.3)	2 (1)
The treatment of sarcopenia necessitates a complex approach, requiring the expertise and involvement of specialized healthcare professionals.	8 (3.8)	42 (19.9)	83 (39.3)	57 (27.0)	21 (10.0)

### General knowledge and factors associated with the numbers of corrected answers on sarcopenia among trainee physicians

The total scores and the distribution of the scores on each topic are demonstrated in **Table 3**. The median total score was 22 out of 30 (73.3%). The highest score of the correct answers was in the area of “etiology” (75%), followed by “terminology and importance” (70%), “diagnosis” (62.5%), and “management and prevention” (62.5%). Factors associated with the numbers of corrected answers using stepwise forward multiple regression analysis with logistic transformation are shown in **Table 4**. The lower age of the trainee physicians, increased years of practice, and training in the “internal medicine group” compared with other specialists affected the sarcopenia knowledge scores.

**Table 3.** Total score on sarcopenia knowledge

**Topics**	**No. of correct answers; median (IQR 1, 3)**	**No. of items**	**(%); median (IQR 1, 3)**
A. Terminology and importance	7 (6, 8)	10	70 (60, 80)
B. Etiology	3 (3, 4)	4	75 (74, 100)
C. Diagnosis	5 (3, 6)	8	62.5 (37.5, 75)
D. Management and prevention	5 (5, 6)	8	62.5 (62.5, 75.0)
Total score	22 (18, 24)	30	73.3 (60, 80)

**Table 4.** Factors associated with higher scores of sarcopenia knowledge using stepwise forward multiple regression analysis with logistic transformation

**Factors**	**Adjusted OR**	**95% CI**	***P***
Age (years)	0.5	(0.4, 0.7)	<0.05^*^
Years of practice	2.0	(1.3, 3.1)	<0.05^*^
Working place			
Internal medicine	11.5	(2.5, 53.4)	<0.05^*^
Family medicine	7.8	(0.4, 169.3)	0.19
Others	1	−	−
Clinical experience in nutrition	4.3	(0.7, 26.0)	0.11

* *P* < 0.05.

CI, confidence interval; OR, odds ratio.

## Discussion

The findings of this survey indicated that the trainee physicians were familiar with the term “sarcopenia,” but lacked certainty in the identification and management of this condition. Their attention was primarily directed toward specific diseases rather than sarcopenia. In comparison to previous studies conducted in the United States and the Netherlands, they revealed that physicians exhibited varying degrees of familiarity with sarcopenia depending on their specialties; however, the majority of them were uncertain about the diagnostic process in a clinical setting [14, 17]. A study involving cancer clinicians, including dietitians, nurses, physicians, and other allied health clinicians, demonstrated awareness of the significance of sarcopenia among their patients, yet they expressed less confidence in identifying this condition in individuals [18]. The overall results pertaining to general knowledge of sarcopenia in this study were favorable, with a score of 22 out of 30. The respondents demonstrated proficiency in the domains of terminology, importance, and etiology, while their performance in diagnosis, management, and prevention was comparatively weaker. These findings align with previous investigations on the subject [14, 15, 17, 19], signifying that despite the substantial growth in research on sarcopenia in recent years, there are specific guidelines available in various regions [1, 10,11,12], and the awareness among physicians regarding this condition is increasing. However, the practical implementation of these guidelines in clinical settings remains limited. An instance of this can be seen in an online investigation carried out on oncologists residing in Australia. This investigation aimed to assess their level of awareness regarding sarcopenia. The findings revealed that a significant majority of the oncologists demonstrated cognizance of this condition, while also recognizing its relevance in their professional responsibilities, particularly in terms of detecting sarcopenia. However, it is noteworthy that <50% of the surveyed oncologists (specifically, 43%) expressed limited confidence when it came to accurately identifying and effectively managing sarcopenia [18]. One longitudinal study conducted among healthcare professionals in the Netherlands revealed that although they were aware of the existence of sarcopenia, only a minority possessed the necessary knowledge to identify and detect sarcopenia [14]. Similarly, another longitudinal study conducted among healthcare professionals in Australia and New Zealand discovered that their understanding of sarcopenia was limited before attending a professional event on the subject. Furthermore, after 6 months, their retention of knowledge experienced significant declines, leading to a limited ability to diagnose sarcopenia as part of their professional practice [15]. In the United Kingdom, a limited number of medical professionals have made the determination of sarcopenia utilizing an established algorithm, with a portion of these individuals relying on medical history instead of empirical evaluations of muscle mass and functionality. Furthermore, the application of an existing therapeutic regimen, consisting of resistance-based exercise, was not consistently executed in patients suspected of having sarcopenia [19].

Increased duration of practice, lower age of the trainee physicians, and being trained in the “internal medicine group” compared with other specialists were the factors that displayed an association with the sarcopenia knowledge scores in this study. The scores were influenced by the increased duration of practice. A potential explanation for this observation is that a greater number of years spent in practice may result in enhanced skills and experience in geriatric conditions, including sarcopenia [20]. On the contrary, a lower age was also found to be linked to higher scores in general knowledge. This can be attributed to the fact that the physicians who applied for the residency training program at the Faculty of Medicine, Khon Kaen University primarily obtained their training from “interns” (those who have completed their 6th year of medical school) rather than “residents” (who generally have at least 3 years of experience as a general practitioner). Additionally, sarcopenia has been introduced during both undergraduate and postgraduate training through bedside teaching and lectures. Therefore, younger trainees tend to possess more knowledge about this condition. It was only the specialty training in “internal medicine” that resulted in significant increases in the respondents' scores. Physicians specializing in this field are more likely to encounter sarcopenic patients due to the high prevalence of this condition in both inpatient and outpatient settings [21, 22].

The potential rationales encompassed a significant disparity between knowledge and implementation in the realm of clinical practice. The aforementioned factors include a universal guideline, the presence of diagnostic tools, a lack of motivation among physicians to conduct diagnosis and management of sarcopenia, and the insufficiency of treatment options beyond exercise and nutrition [14, 15, 17]. The findings from this investigation suggest that there may be advantages to modifying the educational programs for medical students at both the undergraduate and postgraduate levels to enhance their understanding and perspective on sarcopenia. This could be achieved by incorporating interactive interventions or other novel interventions alongside traditional didactic teaching methods, not solely for undergraduate education but also for postgraduate education encompassing all specialized areas that cater to geriatric patients. Moreover, the attainment of success in establishing a national clinical practice guideline for screening, diagnosing, and managing sarcopenia, which encompasses the utilization of diagnostic tools in clinical settings, is contingent upon the collaboration and support of healthcare professionals from health professional associations [15, 17].

The significant advantage of this research resides in its position as the first study to evaluate the attitude and knowledge levels among physicians in Thailand. Nonetheless, it is imperative to acknowledge that the generalizability of the results may be limited, given that the current participants comprised young physicians undergoing a training program, and due to the relatively limited sample size in this study, we were unable to conduct a subgroup analysis. Furthermore, the potential for selection bias might be present in this study, since the utilization of an online self-report questionnaire may have triggered socially desirable responses. As demonstrated, the response rates were comparatively lower among trainees specializing in fields other than internal medicine, thereby suggesting a diminished emphasis on the identification and treatment of sarcopenia in their respective clinical practices. Further research utilizing a multicenter framework is projected to generate deeper and more informative findings.

In conclusion, the term “sarcopenia” was known by most of the trainee physicians, although their confidence in detecting, preventing, and managing this condition was lacking. In terms of knowledge, the trainee physicians generally performed well, but only to a moderate extent in the areas of diagnosis, prevention, and management, which corresponds to their attitudes. Certain factors, such as the duration of practice, the younger age of the trainee physicians, and a specialization in internal medicine, were significantly associated with higher scores on knowledge. These findings highlight the necessity of enhancing undergraduate and postgraduate training by incorporating sarcopenia into medical curricula. Moreover, it is recommended that policymakers and healthcare professionals collaborate to develop accessible clinical practice guidelines. Conducting future surveys would be advantageous for monitoring trends in attitude and knowledge of sarcopenia in geriatric patients.

## References

[1] Chen LK, Woo J, Assantachai P, Auyeung TW, Chou MY, Iijima K (2020). Asian Working Group for Sarcopenia: 2019 consensus update on sarcopenia diagnosis and treatment. J Am Med Dir Assoc.

[2] Cho MR, Lee S, Song SK (2022). A review of sarcopenia pathophysiology, diagnosis, treatment and future direction. J Korean Med Sci.

[3] Sayer AA, Cruz-Jentoft A (2022). Sarcopenia definition, diagnosis and treatment: consensus is growing. Age Ageing.

[4] Yuan S, Larsson SC (2023). Epidemiology of sarcopenia: prevalence, risk factors, and consequences. Metabolism.

[5] Brugliera L, Giordani A, D'Angelo G, Trimarchi C, Villa G, Yen TY (2023). Prevalence of sarcopenia in older patients in rehabilitation wards. J Pers Med.

[6] Cervera-Díaz MDC, López-Gómez JJ, García-Virto V, Aguado-Hernández HJ, De Luis-Román DA (2023). Prevalence of sarcopenia in patients older than 75 years admitted for hip fracture. Endocrinol Diabetes Nutr (Engl Ed).

[7] Zuo X, Li X, Tang K, Zhao R, Wu M, Wang Y (2023). Sarcopenia and cardiovascular diseases: a systematic review and meta-analysis. J Cachexia Sarcopenia Muscle.

[8] Knoedler S, Schliermann R, Knoedler L, Wu M, Hansen FJ, Matar DY (2023). Impact of sarcopenia on outcomes in surgical patients: a systematic review and meta-analysis. Int J Surg.

[9] Jang JY, Kim D, Kim ND (2023). Pathogenesis, intervention, and current status of drug development for sarcopenia: a review. Biomedicines.

[10] Baek JY, Jung HW, Kim KM, Kim M, Park CY, Lee KP (2023). Korean Working Group on Sarcopenia Guideline: expert consensus on sarcopenia screening and diagnosis by the Korean Society of Sarcopenia, the Korean Society for Bone and Mineral Research, and the Korean Geriatrics Society. Ann Geriatr Med Res.

[11] Wallengren O, Bosaeus I, Frändin K, Lissner L, Falk Erhag H, Wetterberg H (2021). Comparison of the 2010 and 2019 diagnostic criteria for sarcopenia by the European Working Group on Sarcopenia in Older People (EWGSOP) in two cohorts of Swedish older adults. BMC Geriatr.

[12] Zanker J, Sim M, Anderson K, Balogun S, Brennan-Olsen SL, Dent E (2023). Consensus guidelines for sarcopenia prevention, diagnosis and management in Australia and New Zealand. J Cachexia Sarcopenia Muscle.

[13] Yao XM, Liu BB, Deng WY, Wang XH (2022). The awareness and knowledge regarding sarcopenia among healthcare professionals: a scoping review. J Frailty Aging.

[14] Reijnierse EM, de van der Schueren MAE, Trappenburg MC, Doves M, Meskers CGM, Maier AB (2017). Lack of knowledge and availability of diagnostic equipment could hinder the diagnosis of sarcopenia and its management. PLoS One.

[15] Yeung SSY, Reijnierse EM, Trappenburg MC, Meskers CGM, Maier AB (2020). Current knowledge and practice of Australian and New Zealand health-care professionals in sarcopenia diagnosis and treatment: time to move forward!. Australas J Ageing.

[16] Khuankaew K, Limpawattana P, Manjavong M, Saengwijit N, Ojongpien K, Tanlawan P (2024). Nurses' perspectives and understanding of sarcopenia in a tertiary care hospital. J Aging Res.

[17] Guralnik JM, Cawthon PM, Bhasin S, Fielding R, Magaziner J, Cruz-Jentoft AJ (2023). Limited physician knowledge of sarcopenia: a survey. J Am Geriatr Soc.

[18] Kiss N, Bauer J, Boltong A, Brown T, Isenring L, Loeliger J (2020). Awareness, perceptions and practices regarding cancer-related malnutrition and sarcopenia: a survey of cancer clinicians. Support Care Cancer.

[19] Offord NJ, Clegg A, Turner G, Dodds RM, Sayer AA, Witham MD (2019). Current practice in the diagnosis and management of sarcopenia and frailty – results from a UK-wide survey. J Frailty Sarcopenia Falls.

[20] Limpawattana P, Worawittayakit K, Paopongpaiboon P, Chotmongkol V, Manjavong M, Sawanyawisuth K (2018). How nurses understand and perceive delirium in older adults?. J Med Assoc Thai.

[21] Anani S, Goldhaber G, Brom A, Lasman N, Turpashvili N, Shenhav-Saltzman G (2020). Frailty and sarcopenia assessment upon hospital admission to internal medicine predicts length of hospital stay and re-admission: a prospective study of 980 patients. J Clin Med.

[22] Martínez-Calvache V, Herrera-Peña ÁM, Carrera-Gil FJ (2020). Sarcopenia and frailty in older adults hospitalized in internal medicine wards. Acta Medica Colomb.

